# *Mycobacterium tuberculosis* WhiB1 represses transcription of the essential chaperonin GroEL2

**DOI:** 10.1016/j.tube.2012.03.001

**Published:** 2012-07

**Authors:** Melanie R. Stapleton, Laura J. Smith, Debbie M. Hunt, Roger S. Buxton, Jeffrey Green

**Affiliations:** a Department of Molecular Biology and Biotechnology, University of Sheffield, Sheffield S10 2TN, UK; b Division of Mycobacterial Research, MRC National Institute for Medical Research, Mill Hill, London NW7 1AA, UK

**Keywords:** Chaperone, Cmr, Iron–sulfur protein, TB, Transcription regulation, WhiB-like protein

## Abstract

A central feature of TB pathogenesis is the formation of *Mycobacterium tuberculosis* latent infections that can persist for decades. Nitric oxide produced by infected lung macrophages promotes expression of genes associated with dormancy, and impaired nitric oxide production can lead to reactivation of latent disease. Recently, WhiB1 was identified as a nitric oxide-responsive transcription factor. Here it is shown that apo-WhiB1 binds to *groEL2* (*Rv0440*) promoter DNA. Apo-WhiB1 inhibited transcription from the *groEL2* promoter *in vitro* and the transcript start was located ∼181 bases upstream of the *groEL2* start codon. Electrophoretic mobility shift assays with sub-fragments of the *groEL2* promoter indicated that the complete *Rv0439c-Rv0440* intergenic region was required for WhiB1 binding, suggesting that this region possessed more than one WhiB1-binding site. DNase I footprinting identified a WhiB1-binding region that overlapped the −35 element of the *groEL2* promoter. The CRP-family transcription factor Cmr (Rv1675c) was shown to bind the *groEL2* promoter and activate transcription *in vitro* in the presence or absence of cAMP. Therefore, it is suggested that WhiB1 acts to oppose Cmr-mediated cAMP-independent activation of *groEL2* expression in the presence of nitric oxide by promoter occlusion.

## 1 Introduction

The etiological agent of tuberculosis, *Mycobacterium tuberculosis*, causes the deaths of two million people annually.^1^ Its efficiency as a pathogen is partially due to the ability to adapt to the disparate environments encountered during the process of infection. The preferred niche for *M. tuberculosis* is the lung macrophage where it is exposed to reactive oxygen species (e.g. superoxide), reactive nitrogen species (e.g. nitric oxide), low pH, toxic peptides and fatty acids, hypoxia and essential element starvation.^2^ During transmission, *M. tuberculosis* must cope with the stresses (e.g. low temperature, dehydration) associated with residence in the droplet nuclei that are expelled from an infected host. Adaptation to these changing environments requires the reprogramming of *M. tuberculosis* gene expression coordinated by ∼190 transcription regulators responding to diverse signals.^3^ Recently, *M. tuberculosis* WhiB1 (a member of the WhiB-like (Wbl) protein family) was shown to be a nitric oxide-responsive transcription factor.^4–7^ Exposure of *M. tuberculosis* to nitric oxide initiates the dormancy gene expression program that may contribute to the establishment of latent TB infections.^8^ Hence the influence of WhiB1 on *M. tuberculosis* gene expression is potentially significant in TB pathogenesis. Several states of the WhiB1 protein have been identified. The [4Fe–4S] form of WhiB1 (holo-form) was incapable of binding at the *whiB1* promoter, whereas the reduced and oxidized forms of apo-WhiB1, as well as nitric oxide-treated holo-WhiB1 (nitrosylated-form) were able to specifically bind DNA.^7^ Thus, the presence or absence and state of the WhiB1 iron–sulfur cluster (nitrosylated or non-nitrosylated) as well as the redox state of apo-WhiB1 influence the ability to bind DNA and regulate transcription.^7^ It is not yet clear whether the different DNA-binding forms of WhiB1, oxidized and reduced apo-WhiB1 and nitrosylated WhiB1, elicit the same transcriptional responses or recognize the same DNA targets. Nevertheless, because the *M. tuberculosis whiB1* gene is essential, the influence of this regulator in the reprogramming of gene expression is of considerable interest.^7^ However, until the present work, the only recognized WhiB1 target was the *whiB1* promoter itself.^7^ Here a second WhiB1-regulated gene is identified; *groEL2*.

*M. tuberculosis* has two chaperonin genes: *groEL1* (*Rv3417c*) is located downstream of *whiB3* (*Rv3416*), which, like *whiB1*, encodes a member of the Wbl protein family; and *groEL2* (*Rv0440*). The *groEL1* gene is dispensable, whereas *groEL2* is essential.^9^ The paradigm for chaperonin function is the sequestration of unfolded or mis-folded proteins in a chamber formed from two stacked heptameric rings of GroEL capped by GroES where refolding can occur in a process that requires ATP hydrolysis.^10^ However, the isolated GroEL2 protein from *M. tuberculosis* does not form such a structure but is dimeric and lacks ATPase activity.^11^ Furthermore, the GroEL2 protein is highly antigenic and modulates the immune environment by stimulating the release of the cytokines interleukin-10 and tumor necrosis factor-α from monocytes in a CD14-independent manner.^12^ Thus, the *M. tuberculosis* GroEL2 chaperonin deviates significantly from the established paradigm.^13^

Expression of *groEL2* was increased upon heat shock in a process involving the repressor protein HrcA, and lowered in response to Mg(II)-starvation and in a *cmr* (*Rv1675c*) mutant 2 h into macrophage infection; *cmr* encodes a member of the cyclic-AMP receptor protein (CRP) family of transcription factors that is proposed to regulate cAMP-induced genes in macrophages.^14–17^ Here the nitric oxide-responsive transcription regulator WhiB1 is identified as a repressor of *groEL2* expression.

## 2 Materials and methods

### 2.1 Isolation of proteins

WhiB1 and *Mycobacterium smegmatis* RNA polymerase were isolated as described previously.^7^ Where indicated WhiB1 was treated with nitric oxide (20:1 molar ratio of nitric oxide:WhiB1 for 10 min at 20 °C) to activate DNA-binding. The Cmr (Rv1675c) protein was overproduced with a N-terminal hexa-His-tag in *Escherichia coli* from plasmid (pGS2103; a pET28a derivative) and isolated by affinity chromatography on a 1 ml Hi-Trap Chelating column (GE Healthcare) using the manufacturer's standard protocol.

### 2.2 Electrophoretic mobility shift assays and *in vitro* transcription reactions

Electrophoretic mobility shift assays (EMSA) were as described previously.^7^ Radiolabeled *Rv0439c-Rv0440* intergenic DNA (*groEL2*) or fragments thereof, or *ahpC* promoter DNA (∼1.6 nM) were incubated with 0–40 μM His_6_-WhiB1 in the presence of 40 mM Tris pH 8.0, 1 mM EDTA, 100 mM NaCl, 1 mM DTT, 10 mM MgCl_2_, 0.25 mg ml^−1^ bovine serum albumin and 1 μg calf thymus DNA, for 5 min on ice. For the analysis of Cmr–DNA interactions, the protein (0–8 μM) was pre-incubated with the *groEL2* promoter DNA for 10 min at 25 °C in the same buffer as above in the presence or absence of 1 mM cAMP. The resulting complexes were then separated on 6% polyacrylamide gels. *In vitro* transcription reactions were assembled and quantified as described previously except that the indicated fragments of the *Rv0439c-Rv0440* (*groEL2*) intergenic region were used.^7^

### 2.3 DNase I footprinting

Radiolabeled *Rv0439c-Rv0440* (*groEL2*) intergenic region (∼60 ng) was incubated with 20 μM His_6_-WhiB1 in the presence of 40 mM Tris pH 7.5, 50 mM NaCl, 10 mM MgCl_2_, 0.5 mM EDTA, 1 mM DTT and 0.25 mg ml^−1^ bovine serum albumin for 10 min on ice. The complexes were then digested with 1 unit of DNase I for 15–60 s at 25 °C. Reactions were stopped by the addition of 200 μl 0.3 M sodium acetate (pH 5.2) containing 20 mM EDTA, followed by phenol/chloroform extraction. The DNA was ethanol-precipitated and resuspended in loading buffer (80% v/v formamide, 0.1% w/v SDS, 10% v/v glycerol, 8 mM EDTA, 0.1% w/v bromophenol blue, 0.1% w/v xylene cyanol) for electrophoretic fractionation on 6% polyacrylamide–urea gels and autoradiographic analysis. Maxam and Gilbert G tracks of the DNA fragments were used to provide a calibration.^18^

## 3 Results

### 3.1 Apo-WhiB1 binds at the *Rv0439c-groEL2* intergenic region

**Figure 1:**
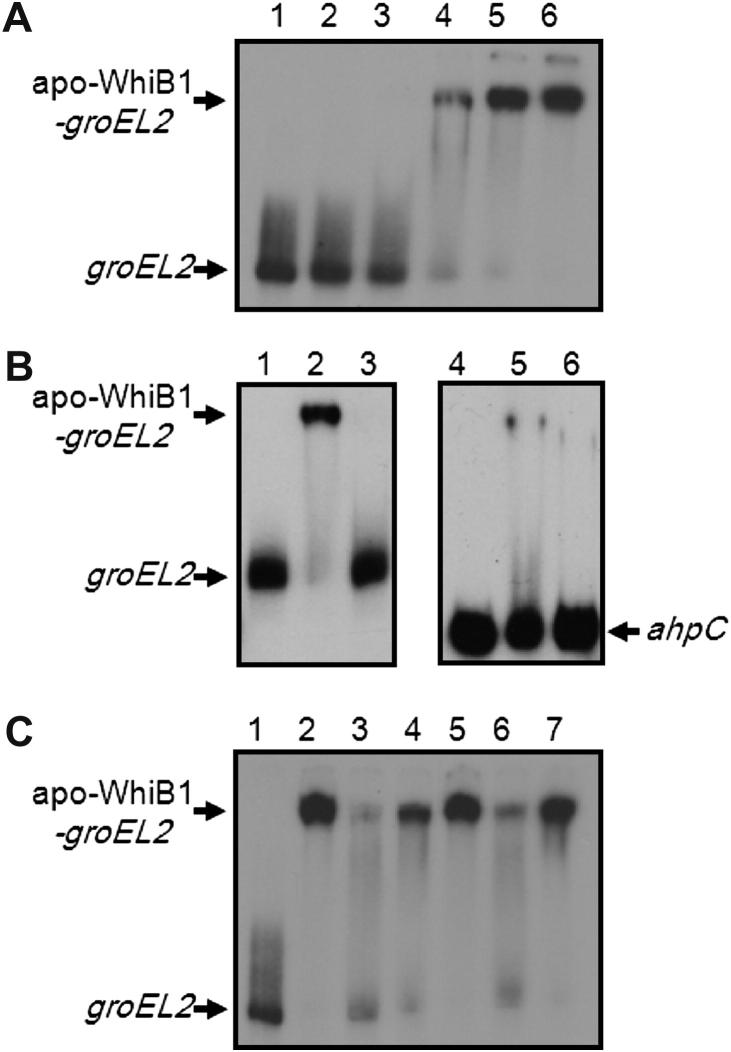
WhiB1 binds at *Rv0439c-Rv0440* (*groEL2*) intergenic region. (A) Radiolabeled *Rv0439c-Rv0440* (*groEL2*) intergenic DNA was incubated with increasing concentrations of apo-WhiB1 before separation of protein-DNA complexes by electrophoresis. Lane 1, no protein; lanes 2–6 contain, 2.5, 5, 10, 20 and 40 μM apo-WhiB1, respectively. (B) The iron–sulfur form of WhiB1 (holo-WhiB1) does not bind at the *Rv0439c-Rv0440* (*groEL2*) intergenic region. Lane 1, no protein; lane 2, apo-WhiB1 (20 μM); lane 3, holo-WhiB1 (20 μM). WhiB1 does not bind at the *ahpC* promoter. Lane 4, no protein; lane 5, apo-WhiB1 (20 μM); lane 6, holo-WhiB1 (20 μM). (C) Apo-WhiB1 binding to radiolabeled *Rv0439c-Rv0440* (*groEL2*) DNA is inhibited by unlabeled *Rv0439c-Rv0440* (*groEL2*) DNA but not by unlabeled *rpfA* promoter DNA. Lane 1, no protein; lanes 2–7 apo-WhiB1 (20 μM) in the absence (lanes 2 and 5), or presence of 100- and 50-fold molar excess unlabeled *Rv0439c-Rv0440* (*groEL2*) DNA (lanes 3 and 6) or unlabeled *rpfA* promoter DNA (lanes 4 and 7). The locations of the free DNA (*groEL2*, *ahpC*) and the DNA-apo-WhiB1 complex (apo-WhiB1-*groEL2*) are indicated.

Several *M. tuberculosis* promoter regions were tested as targets for regulation by apo-WhiB1. Binding was detected in electrophoretic mobility shift assays (EMSA) when the *Rv0439c-Rv0440* (*groEL2*) intergenic region was the target (Figure 1A). As previously observed with the *whiB1* promoter itself (Ref. 7), binding at the *Rv0439c-Rv0440* (*groEL2*) intergenic region saturated over a narrow range of apo-WhiB1 concentration, consistent with cooperative interactions with the DNA, and implying the presence of more than one apo-WhiB1 binding site. Nevertheless, the apo-WhiB1 interactions were judged to be specific because binding was not detected when the *ahpC* promoter was used (Figure 1B, lanes 4–6) or the *rpfA* promoter (Ref. 7), and whereas the formation of the apo-WhiB1 complex with radiolabeled *Rv0439c-Rv0440* (*groEL2*) intergenic DNA was inhibited by excess (100- and 50-fold) unlabeled *Rv0439c-Rv0440* (*groEL2*) competitor DNA (Figure 1C, lanes 3 and 6) this was not so when unlabeled *rpfA* DNA was the competitor (Figure 1C, lanes 4 and 7). Furthermore, the iron–sulfur holo-form of WhiB1 did not bind *Rv0439c-Rv0440* (*groEL2*) intergenic DNA (Figure 1B, lane 3). It was therefore concluded that apo-WhiB1 specifically binds at the *Rv0439c-Rv0440* (*groEL2*) intergenic region and that binding was modulated by the presence/absence of the iron–sulfur cluster.

### 3.2 Apo-WhiB1 inhibits transcription of *groEL2*

**Figure 2:**
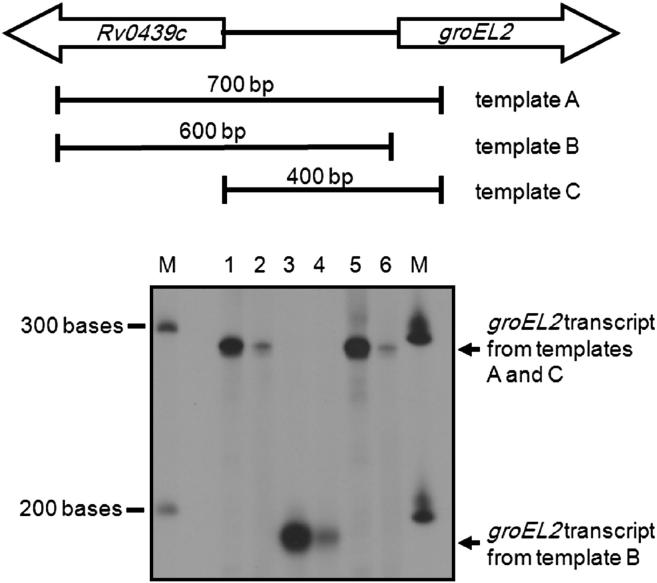
Apo-WhiB1 inhibits transcription of *groEL2 in vitro*. Reactions in lanes 1, 3 and 5 contained: 0.1 pmole of template DNA (lane 1, template A; lane 3, template B; lane 5, template C, as shown in the upper panel), 1 pmole *M. smegmatis* RNA polymerase, 40 mM Tris–Cl pH 8.0, 10 mM MgCl_2_, 70 mM NaCl, 1 mM EDTA, 1 mM DTT, 250 μg ml^−1^ bovine serum albumin, 5% glycerol. Reactions in lanes 2 (template A), 4 (template B) and 6 (template C) were pre-incubated with apo-WhiB1 (20 μM) for 10 min at 37 °C. The sizes of the standard RNA molecules (lanes M) used to calibrate the gel are indicated.

**Figure 3:**
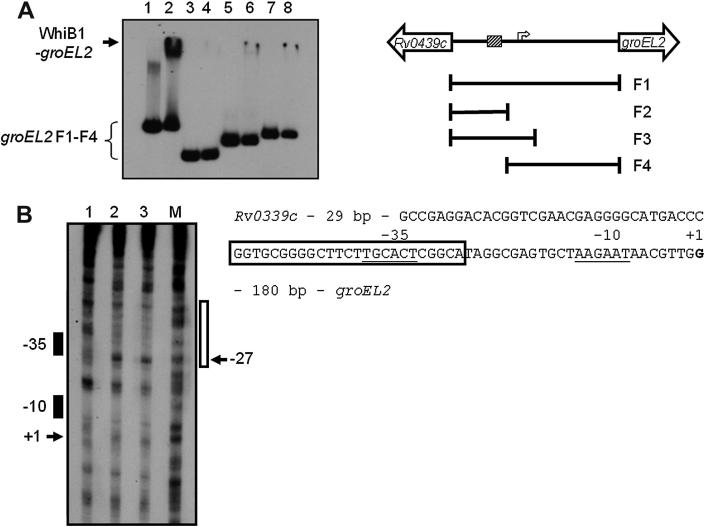
Apo-WhiB1 binds at a site overlapping the −35 element of the *groEL2* promoter. (A) Radiolabeled fragments of *Rv0439c-Rv0440* (*groEL2*) intergenic DNA (F1–4) were incubated with nitric oxide-treated WhiB1 before separation of protein-DNA complexes by electrophoresis. The DNA fragments are shown on the right. The transcript start (arrow) and region protected by apo-WhiB1 in DNase I footprints (hatched box) are indicated. Lanes 1 and 2, *Rv0439c-Rv0440* (*groEL2*) intergenic region extending from −116 to +181 bp (F1); lanes 3 and 4, *Rv0439c-Rv0440* (*groEL2*) sub-fragment extending from −116 to –16 bp (F2); lanes 5 and 6, *Rv0439c-Rv0440* (*groEL2*) sub-fragment extending from −116 to +34 bp (F3); lanes 7 and 8, *Rv0439c-Rv0440* (*groEL2*) sub-fragment extending from −15 to +181 bp (F4). Numbering is relative to the *groEL2* transcript start. Lanes 1, 3, 5 and 7, no protein; lanes 2, 4, 6 and 8, nitric oxide-treated WhiB1 (20 μM). The locations of the free DNA species (F1–F4) and the WhiB1 complexes (WhiB1-*groEL2*) are indicated. (B) DNase I footprint of the *groEL2* promoter: lane 1, no apo-WhiB1, lanes 2 and 3, apo-WhiB1 (20 μM), lane M, Maxam and Gilbert G track. The region of DNA protected from DNase I digestion is indicated by the open rectangle; a hypersensitive site (−27) is arrowed. The closed rectangles indicate the locations of the −35 and −10 elements and the transcript start is marked by +1. The DNA sequence of the *groEL2* promoter is shown on the right. The region of apo-WhiB1 protection is boxed, the −35 and −10 elements are underlined and the transcript start (+1) is in bold type.

*In vitro* transcription assays were used to determine the consequences of apo-WhiB1 binding at the *Rv0439c-Rv0440* (*groEL2*) intergenic region. Three templates were designed to distinguish which of the divergent *Rv0439c* and *groEL2* promoters was active and potentially regulated by WhiB1 (Figure 2). If the *Rv0439c* promoter was active, templates A and B would yield the same sized product and template C would yield a product 300 bases smaller than templates A and B. If the *groEL2* promoter was active then templates A and C would yield the same sized product and template B would yield a product 100 bases smaller than that produced by templates A and C (Figure 2). The experiments showed that templates A and C yielded transcripts of ∼290 bases, and the template C transcript was ∼190 bases (Figure 2). Thus it was concluded that the *groEL2* promoter is active in the presence of *M. smegmatis* σ^A^-RNA polymerase. Furthermore, the sizes of the products identified the location of the *groEL2* transcript start point at ∼181 bases upstream of the start codon, a position associated with potential σ^A^ −10 (AAGAAT, 4/6 matches to the consensus TATAMT) and −35 elements (TGCACT, 4/6 matches to the consensus TTGACW) separated by the optimal 17 bp (Figure 3).^2^ The yield of product from all three transcripts was severely reduced (∼10-fold) when the *in vitro* transcription reactions contained apo-WhiB1 (Figure 2, lanes 2, 4 and 6). Therefore, it was concluded that WhiB1 represses transcription of *M. tuberculosis groEL2*.

### 3.3 DNase I footprinting suggests that apo-WhiB1 represses *groEL2* transcription by promoter occlusion

To better define the DNA elements required for apo- and nitrosylated-WhiB1 binding at the *groEL2* promoter, sub-fragments of the *Rv0439c-Rv0440* (*groEL2*) intergenic region were used as the targets in EMSAs. Whilst the mobility of the complete intergenic region (−116 to +181 bp relative to the *groEL2* transcript start) was retarded by both apo- (Figure 1) and nitric oxide-treated WhiB1 (Figure 3A), sub-fragments encompassing −116 to −16, −116 to +34 or −15 to +181 bp relative to the *groEL2* transcript start were not retarded by either apo- (not shown) or nitric oxide-treated WhiB1 (Figure 3A). These data indicate that WhiB1 requires the complete *Rv0439c-Rv0440* intergenic region to form a stable nucleoprotein complex. DNase I footprinting identified one locus of apo-WhiB1 binding at the *groEL2* promoter as a 25 bp region of protection situated between −26 and −50 relative to the transcript start with a hypersensitive site at −27 (Figure 3B). Thus, the apo-WhiB1 footprint at the *groEL2* promoter was located in a similar position to that observed at the *whiB1* promoter, i.e. overlapping the −35 element.^7^ Therefore, the simplest explanation to account for the *in vitro* transcription data is that apo-WhiB1 represses *groEL2* expression by promoter occlusion.

### 3.4 Cmr activates *groEL2* transcription

**Figure 4:**
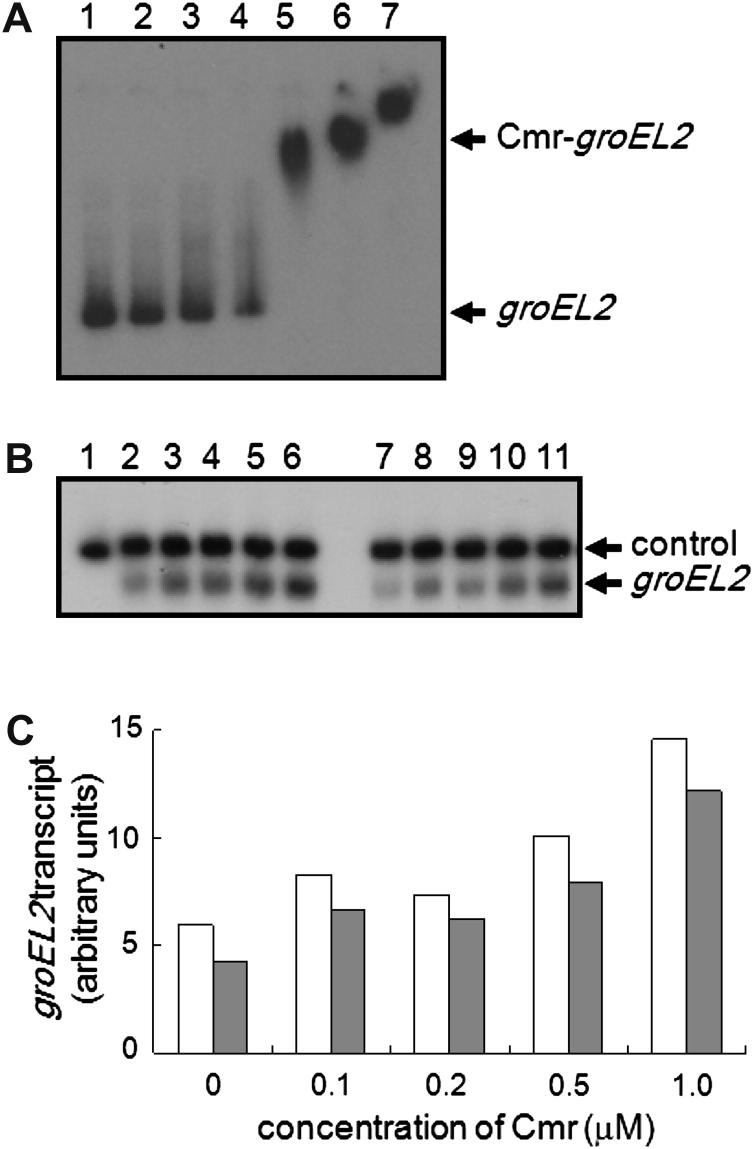
Cmr activates transcription of *groEL2*. (A) Cmr binds at the *groEL2* promoter. Radiolabeled *groEL2* promoter DNA was incubated with increasing concentrations of Cmr (Rv1675c) before separation of protein-DNA complexes by electrophoresis. Lane 1, no protein; lanes 2–7 contain, 0.25, 0.5, 1.0, 2.0, 4.0 and 8.0 μM Cmr, respectively. (B) Cmr activates *groEL2* transcription *in vitro*. A representative autoradiograph is shown. Lane 1, 200 base RNA marker. *In vitro* transcription reactions contained 0.1 pmole of *groEL2* promoter, 1 pmole *M. smegmatis* RNA polymerase, 40 mM Tris–Cl pH 8.0, 10 mM MgCl_2_, 70 mM NaCl, 1 mM EDTA, 1 mM DTT, 250 μg ml^−1^ bovine serum albumin, 5% glycerol with increasing amounts of Cmr: lanes 2 and 7, 0 μM; lanes 3 and 8, 0.1 μM; lanes 4 and 9, 0.2 μM; lanes 5 and 10, 0.5 μM; and lanes 6 and 11, 1.0 μM. Reactions in lanes 7–11 contained 1 mM cAMP. The *groEL2* transcript and the loading control are indicated. (C) The amounts of *groEL2* transcript, in the presence (filled bars) or absence (open bars) of 1 mM cAMP, in each of the reactions shown in (B) were quantified using ImageMaster software (GE Healthcare) and plotted as a histogram. Typically 1.0 μM Cmr resulted in ∼3-fold activation of *groEL2* transcription over the basal level.

*M. tuberculosis* Cmr (Rv1675c) is a member of the CRP family of transcription factors that is required for cAMP-induced protein expression, including GroEL2, in macrophages.^16^ This suggested that Cmr activates *groEL2* expression. Binding of Cmr to the *groEL2* promoter was shown by Gazdik et al. (Ref. 16) and was confirmed here (Figure 4A). Cmr binding at the *groEL2* promoter was unaffected by the addition of 1 mM cAMP (not shown). Transcription of *groEL2 in vitro* was enhanced ∼3-fold in the presence of 1 μM Cmr and this activation was independent of cAMP (Figure 4B and C). Activation of *groEL2* transcription by Cmr is consistent with the previously observed down-regulation of *groEL2* expression when the *cmr* mutant was allowed to infect macrophages for 2 h.^16^

## 4 Discussion

Until this report the only other known target for the nitric oxide-responsive transcription factor WhiB1 was its own promoter.^7^ Here it is shown that WhiB1 represses *groEL2* expression. The GroEL2 protein is an essential chaperonin.^9^ Down-regulation of *groEL2* expression by WhiB1 in the presence of nitric oxide should therefore inhibit the growth of *M. tuberculosis* perhaps assisting entry into the dormant state. Expression of *groEL2* is modulated by the transcription factor Cmr (Rv1675c) and is also induced by cAMP.^16,17^ The *in vitro* transcription reactions reported here show that Cmr activates *groEL2* transcription in the absence of cAMP, suggesting that Cmr does not directly mediate the cAMP effect on *groEL2* expression. Like the *groEL2* promoter, expression of the other known target for WhiB1, *whiB1*, responds to cAMP.^19,20^ In the latter case, the mycobacterial CRP protein (Rv3676) acts as a dual regulator of *whiB1* expression in response to cAMP, but activation by cAMP-CRP is inhibited by apo-WhiB1, i.e. WhiB1 negatively auto-regulates expression.^7,20^ Hence it is possible that the cAMP-mediated effects on *groEL2* expression observed *in vivo* arise, at least in part, from CRP-mediated regulation of *whiB1*. Nevertheless, it is clear that both of the WhiB1 targets identified so far are linked to cAMP-signaling. It has recently been shown that upon infection of macrophages, mycobacterium-derived cAMP promotes bacterial survival by subverting host signaling pathways.^21^ Moreover, cAMP is important in *M. tuberculosis* gene regulation.^17,19,20^ The interaction between cAMP-responsive regulators and the nitric oxide-responsive WhiB1 protein might provide a mechanism to integrate the transcriptional response to two important signals associated with infection. Furthermore, although only two promoters (*whiB1* and *groEL2*) have thus far been identified as WhiB1 targets both exhibited cooperative binding to sites overlapping the −35 elements of the promoters, resulting in repression of transcription. These observations suggest that promoter occlusion might be a common feature of gene regulation by WhiB1. However, it is apparent that apo-WhiB1 interactions with the *groEL2* promoter region are complex and the complete *Rv0439c-Rv0440* intergenic region is required to form a stable nucleoprotein complex. This suggests the presence of at least two WhiB1-binding sites within this region and that both the site overlapping the −35 element, that is identified here, and unidentified site(s) are required for detectable WhiB1 binding in EMSAs. Protein:DNA interactions of this type are not unprecedented but usually at least two regulator:DNA complexes are observed in EMSAs. These complexes result from binding of the regulator first to a high affinity site and then subsequent cooperative occupation of a low affinity site. In such cases impairment of the high affinity site abolishes binding to the low affinity site.^22^ It appears that detectable WhiB1 binding to the *Rv0439c-Rv0440* intergenic region is dependent on sites of similar affinity and that binding is highly cooperative.

In conclusion, the work described here reveals new aspects of the regulation of *M. tuberculosis groEL2*. Previously, expression of *groEL2* was shown to be controlled by HrcA (derepression in response to heat shock), by Cmr (activation in response to an unknown cAMP-related signal), and by Mg(II)-starvation (repression by a PhoPR-independent mechanism).^14–16^ Now it is shown that WhiB1 represses *groEL2* in response to signals – nitric oxide, oxidative stress, iron-starvation – that promote the formation of the DNA-binding apo- and nitrosylated-forms of WhiB1. An understanding of the precise interplay between these regulators and the consequences for *groEL2* expression awaits further detailed biochemical analysis. However, it appears that several signals associated with the process of infection are sensed and transduced by integrated gene regulatory circuits to optimize expression of the essential chaperonin GroEL2.
